# Bone marrow transplant-associated thrombotic microangiopathy without peripheral blood schistocytes: a case report and review of the literature

**DOI:** 10.1186/s40164-018-0106-9

**Published:** 2018-06-22

**Authors:** Eric Wirtschafter, Christine VanBeek, Yuliya Linhares

**Affiliations:** 1 Olive View, UCLA Medical Center, Sylmar, CA USA; 2 AmeriPath, Oklahoma, OK USA; 3 Cedars-Sinai Medical Center, Los Angeles, CA USA

**Keywords:** Allogeneic hematopoietic stem cell transplant (HSCT), Transplant-associated thrombotic microangiopathy (TA-TMA), Thrombotic microangiopathy (TMA), Eculizumab, Case report

## Abstract

**Background:**

Bone marrow transplant-associated thrombotic microangiopathy (TA-TMA) is a relatively frequent but under-recognized and under-treated hematopoietic stem cell transplant (HSCT) complication that leads to significant post-transplant morbidity and mortality. Classic TMA-defining laboratory abnormalities appear at different times in the course of TA-TMA development, with schistocytes often appearing later in the disease course. In some severe TMA cases, schistocytes may be absent due to increased endothelial permeability. Unfortunately, many clinicians continue to perceive the presence of schistocytes as an absolute requirement for TA-TMA diagnosis, which leads to delayed recognition and treatment of this potentially fatal transplant complication.

**Methods:**

Patient chart review and PubMed literature search using the term, “transplant-associated thrombotic microangiopathy”.

**Case presentation:**

A 54-year-old male IgG kappa multiple myeloma underwent a reduced intensity allogeneic HSCT from a 9/10 HLA-matched unrelated donor after conditioning with fludarabine and melphalan. On day + 27, the patient developed acute kidney injury followed by repeated episodes of diarrhea and gastrointestinal bleeding attributed to graft versus host disease (GVHD) and cytomegalovirus (CMV) colitis. Repeated colonic biopsies suggested CMV infection and GVHD. Despite appropriate treatment with antiviral therapy and immunosuppressants, the patient’s condition continued to deteriorate. He experienced concomitant anemia and thrombocytopenia as well as elevated lactate dehydrogenase and low haptoglobin levels, but a TA-TMA diagnosis was not made due to an absence of schistocytes on peripheral smear. The patient expired secondary to uncontrolled gastrointestinal bleeding. A post-mortem analysis of the resection specimen revealed extensive TMA involving numerous arteries and arterioles in the ileal and colonic submucosa as well as in the muscularis propria and deep lamina propria of the mucosa.

**Conclusions:**

TA-TMA can occur in the absence of peripheral blood schistocytes. Our experience underscores the importance of considering the diagnosis of intestinal TA-TMA in patients with refractory post-transplant diarrhea and GI bleeding, even if all classic features are not present.

## Background

Bone marrow transplant-associated thrombotic microangiopathy (TA-TMA) is a relatively frequent but under-recognized and under-treated stem cell transplant complication that leads to significant post-transplant morbidity and mortality. Like the more common atypical hemolytic uremic syndrome (aHUS), it is mediated by excessive activation of the alternative pathway of complement [1, 2]. Mutations in genes responsible for complement activation have been identified at a significantly higher rate in stem cell transplant recipients with TA-TMA as compared to patients without TA-TMA [3]. It is possible that stem cell donor variants of complement system regulatory proteins may contribute to TA-TMA development in the recipient [4]. Exogenous risk factors that have been associated with TA-TMA include high-dose chemotherapy, radiation therapy, unrelated stem cell donor, HLA mismatch, exposure to calcineurin inhibitors with or without concomitant exposure to sirolimus, graft-versus-host disease (GVHD), and infections [5]. TA-TMA is thought to be distinct from aHUS in that higher doses of anti-complement therapy (eculizumab) may be required to control the disease and that anti-complement therapy can sometimes be discontinued after a period of treatment [1, 6].

While TA-TMA is an increasingly recognized entity, efforts to more precisely define its incidence and to standardize the best approach to management have been hampered by variable definitions of the condition (Table 1). In 2005 the blood and marrow transplant clinical trials network proposed a series of TA-TMA diagnostic criteria that included ≥ 2 schistocytes/HPF and concurrent renal and/or neurologic dysfunction without an alternative explanation, among other requirements [7]. In 2007 an international working group proposed a set of criteria that were similar to the 2005 criteria but required ≥ 8 schistocytes/HPF without a specific requirement for renal or neurologic dysfunction [8]. A subsequent study attempted to create a more refined set of diagnostic criteria by combining most elements of the original two criteria while also specifying a need to rule-out disseminated intravascular coagulation [9]. Cho et al. reported an estimated 12.7% incidence of TA-TMA in adults undergoing hematopoietic stem cell transplantation with use of their criteria.

**Table 1:** Summary of TA-TMA diagnostic criteria

Diagnostic criteria	Ho [7]	Ruutu [8]	Cho [9]	Jodele [21]
Schistocytes^a^	✓	✓	✓	✓^b^
Elevated LDH	✓	✓	✓	✓
Renal and/or neurologic dysfunction	✓			✓^c^
Thrombocytopenia		✓	✓	✓
Anemia		✓	✓	✓
Low haptoglobin		✓	✓	
Normal PT/PTT	✓	✓	✓	
Negative Coombs test	✓		✓	
Terminal complement activation				✓

^a^Ho and Cho defined increased schistocytes as ≥ 2/HPF. Ruutu defined ≥ 8/HPF as the threshold suggestive of TA-TMA

^b^Jodele noted that histologic evidence of microangiopathy on a tissue specimen may exist in the absence of peripheral blood schistocytosis and considered this finding sufficient for a diagnosis of TA-TMA even in the absence of other any other suggestive clinical features

^c^Urinalysis protein concentration of ≥ 30 mg/dL and/or hypertension considered more reliable than serum creatinine as evidence of renal dysfunction

A feature of all three of the aforementioned criteria is the requirement of schistocytes in the peripheral blood. Jodele and colleagues suggested an updated set of TMA criteria in 2014 [3], which allow for the diagnosis of TA-TMA based solely on a tissue biopsy with evidence of thrombotic microangiopathy, or based on a constellation of laboratory and clinical markers highly suggestive of TMA. The presence of schistocytes is not required to make a diagnosis of TMA according to the updated criteria.

Of note, classic TMA-defining laboratory abnormalities appear at different times in the course of TA-TMA development, with schistocytes often appearing later in the disease course [3]. In some severe TMA cases, schistocytes may be absent due to increased endothelial permeability, with secondary red cell tissue extravasation [10]. Unfortunately, many clinicians continue to perceive the presence of schistocytes as an absolute requirement for TA-TMA diagnosis, which leads to delayed recognition and treatment of this potentially fatal transplant complication.

Here we describe a case of TA-TMA that occurred without evidence of peripheral blood schistocytes.

## Case presentation

A 54 year-old Caucasian male without significant comorbidities was diagnosed with IgG kappa multiple myeloma in 2005. Initial treatment consisted of doxorubicin, vincristine, and dexamethasone followed by an autologous stem cell transplant (SCT) with melphalan 200 mg/m^2^ conditioning. He remained in remission for 2.5 years, at which time he relapsed and was treated with a series of doublet regimens followed by a second autologous SCT in 2011, with melphalan 200 mg/m^2^ conditioning. He relapsed 4 months after the second transplant and was treated with carfilzomib but quickly progressed. The patient eventually achieved a very good partial response with bendamustine and dexamethasone and underwent reduced intensity conditioning with fludarabine 30 mg/m^2^ on days − 6 to − 2 and melphalan 50 mg/m^2^ on days − 3 to − 2, followed by a 9/10 matched unrelated allogeneic SCT in November 2012. GVHD prophylaxis consisted of sirolimus and tacrolimus starting day − 3 as well as methotrexate on days + 1, 3, 6, and 11. On day + 27 post-transplant the patient developed acute kidney injury (creatinine of 2.6 mg/dL from a baseline of 0.7) that was attributed to calcineurin inhibitor toxicity. The patient was switched to mycophenolate mofetil and corticosteroids for GVHD prophylaxis, with normalization of kidney function. By day + 130 the patient was felt to be in at least very good partial remission based on negative serum protein immunofixation and 99.8% peripheral blood donor chimerism.

On day + 132, the patient returned to the hospital with diarrhea with scant blood. He underwent colonoscopy with biopsy. Histologic analysis demonstrated findings consistent with CMV colitis and GVHD: crypt apoptotic bodies, ulcerations, and CMV inclusions were noted. He was started on ganciclovir, and prednisone was increased from 60 mg daily to 60 mg twice daily. He was discharged 2 weeks later, at which time the platelet count had decreased from 93,000/μL on admission (normal 150,000–450,000/μL) to 29,000/μL. The thrombocytopenia was attributed to a combination of antiviral medication and CMV infection.

He returned to the hospital 1 week later (day + 146) with recurrence of profuse diarrhea with small amounts of blood and associated abdominal cramping. Diarrhea was attributed to worsening GVHD. He was restarted on tacrolimus but continued to have maroon-colored stool output. A colonoscopy was repeated and was notable grossly for pancolitis with scattered ulcerations, ileocecal valve ulceration, mild ileitis, anorectal junction ulcers, and internal hemorrhoids that were not bleeding. The pathology report again suggested CMV infection and GVHD (increased crypt apoptotic bodies, focal erosions, and a positive CMV immunostain). During that admission, a diagnosis of TMA was considered due to a persistently low hemoglobin of approximately 9 g/dL (normal 13–17 g/dL) and thrombocytopenia that persisted in 30,000–50,000/μL range. A peripheral smear at that time showed 2–4 schistocytes/HPF in the setting of a haptoglobin of 22 mg/dL (normal 36–195 mg/dL) and a lactate dehydrogenase (LDH) of 731 U/L (normal < 260 U/L). Overall, the patient met 6 out of 7 of the TA-TMA criteria proposed by Cho et al. His tacrolimus was again discontinued, and he was maintained on a combination of sirolimus (goal level 5–7), mycophenolate mofetil, and steroids for GVHD treatment. At the time of discharge, his hemoglobin was 8.7 g/dL and platelets were 20,000/μL.

On day + 156 the patient returned to the hospital with multiple episodes of bright red blood per rectum. He had diffuse patchy ecchymoses on physical exam. The platelet count was 17,000/μL. A colonoscopy was repeated, and per visual inspection the GVHD was felt to be improved. Hemorrhoidal bleeding was suspected as the cause of the bleeding, and colorectal surgery was consulted. He underwent sclerotherapy for hemorrhoids prior to discharge.

On day + 188 TMA was again considered in the setting of an LDH of 1147 U/L. Sirolimus was discontinued. A peripheral blood smear, however, showed no schistocytes, and haptoglobin returned within low-normal range at 43 mg/dL. TMA was therefore felt to be unlikely. A CT scan of the abdomen and pelvis, performed for another indication, revealed an unexpected finding of pneumatosis intestinalis involving the ascending and transverse colon. This finding was attributed to GVHD and CMV and, as the patient was felt to be relatively asymptomatic, no specific intervention was performed. Sirolimus was restarted given the concerning imaging findings and lack of strong evidence for TMA.

The patient was re-admitted on day + 211, this time with melenic stool. A tagged red blood cell scan identified the ascending colon as a source of bleeding. An angiogram with attempted embolization was unsuccessful due to inability to identify a bleeding vessel. Hemolysis labs were repeated given persistent cytopenias, including a hemoglobin of 7.1 g/dL and a platelet count of 37,000/μL. Haptoglobin was undetectable, and LDH was elevated at 1254 U/L. A repeat peripheral smear demonstrated a normocytic, normochromic anemia without increase in schistocytes. Sirolimus was again discontinued. The patient remained anemic and intermittently refractory to red blood cell transfusions, prompting two additional attempts at angiogram/embolization, neither of which successfully identified a bleeding vessel. A bone marrow biopsy was repeated and revealed a hypocellular marrow without evidence of myeloma relapse.

On day + 217 the patient was transferred to the medical ICU for high volume bloody stool output, a hemoglobin of 6.3, and lightheadedness. The INR was 1.0 with a PTT of 22. A wireless capsule endoscopy and a repeat tagged red blood cell scan were both unsuccessful at identifying a source of bleed. A colonoscopy with biopsy was repeated; 2 visible vessels were identified and clipped. Histologic analysis did not show evidence of persistent GVHD or CMV colitis. An EGD with biopsy revealed no abnormalities on histologic analysis. He was managed supportively with transfusions as needed but then began to develop confusion, agitation, and increasing anger. Psychiatry was consulted for delirium. LDH was 767 U/L, and the haptoglobin was low at < 35 mg/dL. Platelets were 47,000/μL, and the INR was 1.1. A peripheral smear was repeated and did not show schistocytes. An ADAMTS13 level was ordered given recurrent suspicion for TMA, and therapeutic plasma exchange (TPE) was initiated empirically pending that result. The patient’s confusion was noted to improve following TPE, and he completed a total of 5 exchanges. LDH improved to 372 U/L. ADAMTS13 level ultimately returned at 60% (normal > 66%). Shortly thereafter, the patient developed copious hematochezia associated with a drop in hemoglobin from 8.9 to 6.9 g/dL. He was taken urgently to the operating room for visceral angiogram, which did not identify a bleeding vessel. An ileocolectomy was performed, but the patient suffered a cardiac arrest intra-operatively and expired shortly thereafter.

**Fig. 1:**
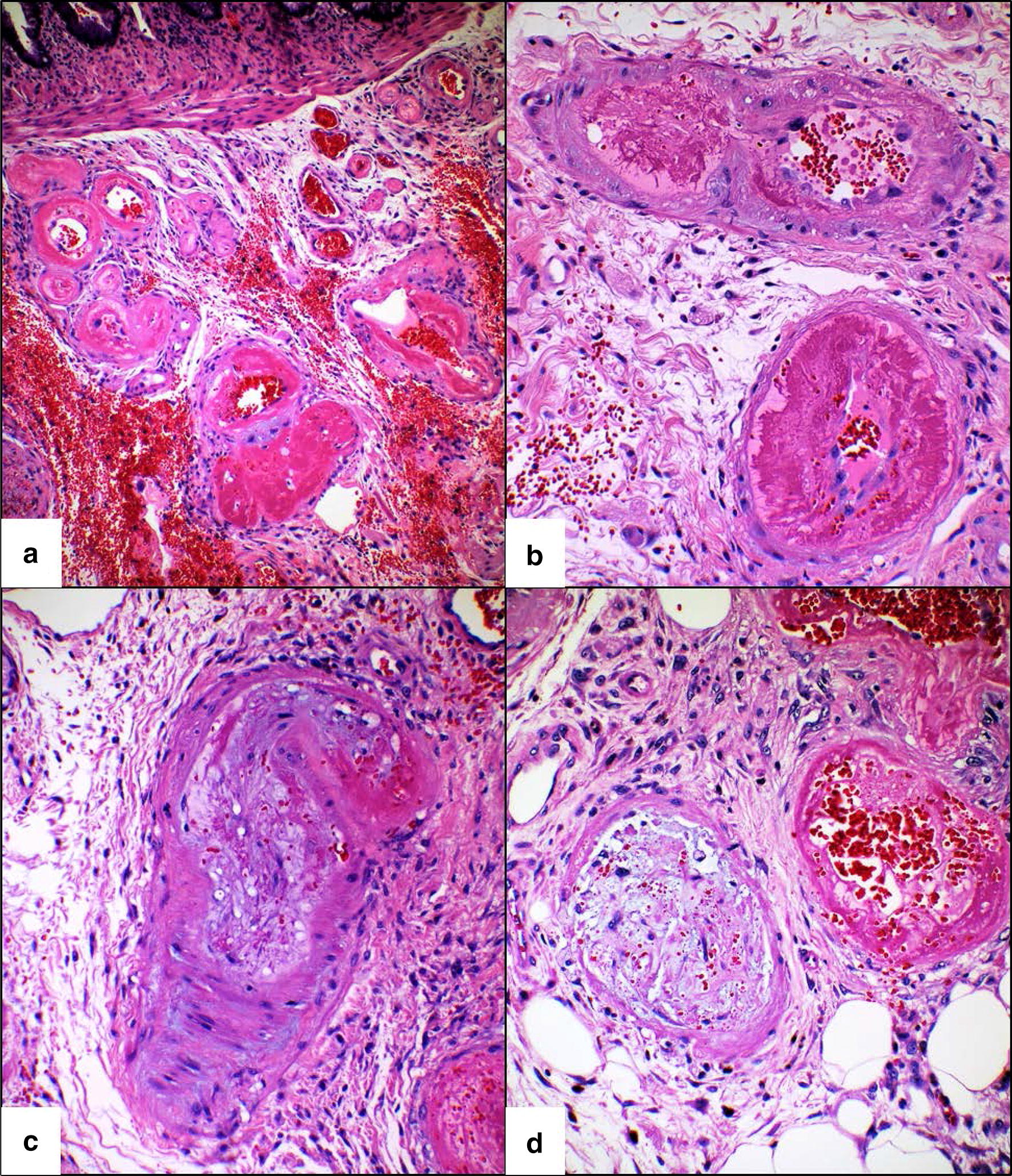
Histologic features of thrombotic microangiopathy in arteries. **a** Several submucosal arteries affected by acute TMA with surrounding perivascular hemorrhage (hematoxylin and eosin, original magnification ×40). **b** Arteries with marked luminal narrowing due to accumulation of intimal fibrin with swelling overlying endothelial cells (hematoxylin and eosin, original magnification ×400). **c** Artery with mark intimal edema. Scattered red cell are present within the artery intimal zone and adjacent perivascular stroma (hematoxylin and eosin, original magnification ×400). **d** Occlusion of arterial lumina due to mucoid intimal edema in left artery and numerous red blood cell fragments with underlying fibrin in right artery (hematoxylin and eosin, original magnification ×400)

**Fig. 2:**
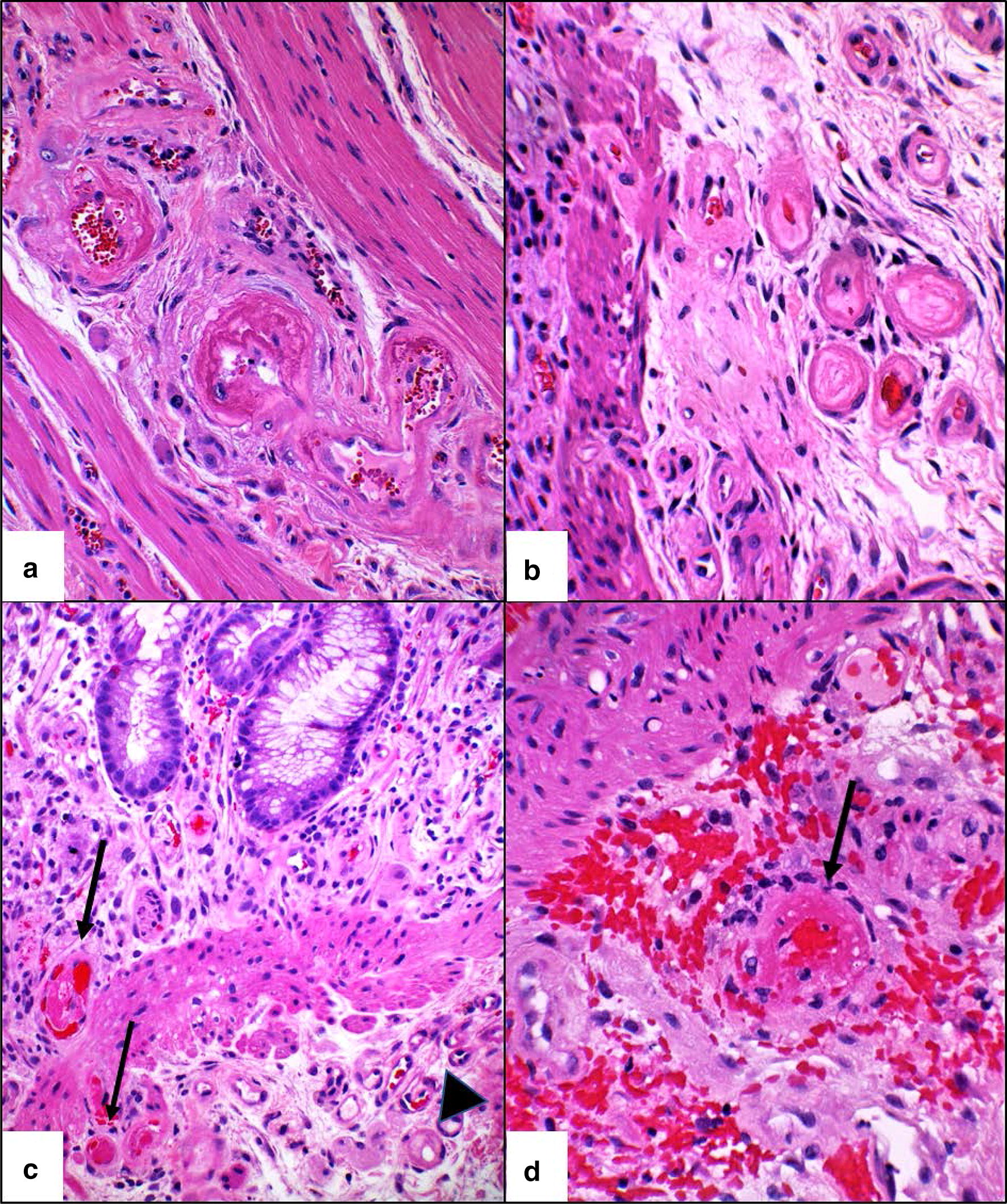
Thrombotic microangiopathy in arterioles of resection specimen (**a**, **b**) and prior colonic mucosal biopsies (**c**, **d**). **a** Arterioles in muscularis propria with subendothelial fibrin. **b** Submucosal arterioles showing marked subendothelial edema with formation of concentric layers of new basement membrane material. **c** Capillaries/arterioles of deep lamina propria and superficial submucosa with intraluminal fibrin thrombi (arrows). Arteriole with subendothelial edema and new subendothelial basement membrane layer (arrowhead). **d** Superficial submucosal arteriole with subendothelial fibrin (arrow) and perivascular hemorrhage. (hematoxylin and eosin, original magnification ×400 for **a**–**d**)

A post-mortem analysis of the resection specimen revealed TMA involving numerous arteries and arterioles in the ileal and colonic submucosa as well as few thrombosed arterioles in the muscularis propria and deep lamina propria of the mucosa (Figs. 1 and 2). Rare thrombosed arterioles were identified in the appendix. The TMA in many of the vessels was active, characterized by endothelial cell swelling, endothelial cell detachment with intimal expansion by pale plasma protein material (“mucoid intimal edema”), and accumulation of fibrin and red cell fragments within expanded intimal zones and vascular lumina. A smaller number of arteries and arterioles showed features of persistent or chronic microangiopathic changes, such as concentric layers of new basement membrane material alternating with intimal edema well as focal presence of foam cells within artery intimal zones. All venous structures were patent without thrombosis. In areas of more severe TMA, there were foci of perivascular hemorrhage and deep ulceration extending into the superficial muscularis propria. Non-ulcerated mucosa showed architectural distortion and crypt regenerative features, consistent with previous injury. Additionally, there were areas of crypt dropout with replacement of mucosa by healing granulation tissue and an overlying layer of regenerative epithelium. Scattered CMV inclusions were visible within endothelial cells of the granulation tissue. However, there was no evidence of CMV infection by light microscopy or immunohistochemistry within endothelium of vessels affected by TMA. There was no significant increase in crypt apoptosis, arguing against GVHD.

A retrospective review of the patient’s previous colonic biopsies was performed. In addition to the initially reported findings, biopsies from days 146 and 217 showed subtle features of TMA (Fig. 2); these included rare thrombosed arterioles in the deep lamina propria and superficial submucosa surrounded by perivascular hemorrhage as well as few arterioles with subendothelial expansion by pale material accompanied by underlying layers of new basement membrane material.

## Discussion

Clinicians must remain vigilant for TA-TMA in the post-transplant setting, as up to 35% of stem cell transplant recipients will experience this complication [11]. The exact time course for presentation of TA-TMA is not well defined, though one early series of 25 patients suggested a median time to presentation of 27 days after transplant (range 17–484 days) [12]. Care must be taken to rule-out conditions that can present with similar clinical and laboratory findings, such as veno-occlusive disease, infection, and GVHD. One recent reports suggests that a serial increase in serum levels of the terminal complement activation complex C5b-9 is a very sensitive predictor of subsequent TA-TMA [13]. While TA-TMA has been most closely linked to allogeneic SCT, it has also been described following autologous SCT as well [14], though this is far less common.

Symptoms of intestinal TA-TMA such as refractory diarrhea and GI bleeding largely overlap with the symptoms of refractory grade 3–4 intestinal GVHD, which makes it difficult to distinguish intestinal TA-TMA (iTMA) from primary or coexisting GI GVHD [15]. Continuous diarrhea and GI bleeding in a patient who has had adequate GI GVHD treatment should prompt consideration of iTMA. In patients with known GI GVHD, persistent bleeding or abdominal pain may suggest co-occurring iTMA: patients with both GI GVHD and iTMA more commonly experience bleeding and pain than patients with iGVHD alone [16]. Additional calcineurin exposure in patients who are treated for refractory GI GVHD but in fact have iTMA can be detrimental, while timely discontinuation of calcineurin inhibitors may lead to TMA reversal [17].

Classic features of thrombotic microangiopathy are not always present in patients with TA-TMA: even in aHUS, renal function may be preserved as often as 20% of the time [18]. Published criteria to-date require schistocytes for a diagnosis of TA-TMA. Importantly, schistocytes are not a finding specific to TA-TMA, having also been linked to infections and GVHD in the post-transplant setting [19]. In the case described here, the patient did not have increased schistocytes in his blood following multiple reviews of the peripheral smear, though the post-mortem studies later clearly identified evidence of TMA. Indirect measures of hemolysis, though not strictly speaking a part of the TA-TMA diagnostic criteria, were similarly inconsistent. Circulating erythroid precursors were seen intermittently on peripheral smear but not in all cases. Absolute reticulocyte counts ranged from 27,000 to 97,000/mm^3^, suggestive of an overall suboptimal bone marrow response to anemia, though interpretation of this laboratory parameter was difficult given the patient’s recent stem cell transplantation. Indirect bilirubin, another marker of hemolysis, was never elevated in this patient. It is likely that the degree of intravascular hemolysis was insufficient to result in bilirubin elevation. Finally, while the LDH was consistently increased, possibly secondary to bowel ischemia due to intestinal TA-TMA, this is a non-specific finding that did not provide diagnostic clarity at the time.

We believe our case represents an occurrence of intestinal TMA secondary to a systemic TA-TMA process but without the classic finding of peripheral blood schistocytes. Intestinal TMA has been described as a distinct subset of TA-TMA and may frequently occur without laboratory evidence of systemic TMA, requiring histologic examination of gastrointestinal (GI) pathology specimens to make a diagnosis. In one retrospective series, 80 out of 886 post-allogeneic transplant patients with diarrhea were found to have histologic evidence of iTMA in GI mucosal biopsies [20]. Of those 80 patients only 46% were reported to have > 2 schistocytes per/HPF in the peripheral blood.

Histologic features of iTMA are similar to those of TMA in any other organ. In mucosal biopsies, the capillaries and arterioles exhibit variable features of endothelial cell injury such as endothelial cell swelling, endothelial cell detachment and new basement membrane formation, intraluminal fibrin and thrombosis, and intraluminal schistocytes. In resection specimens, small to medium sized arteries may also be involved. The TMA leads to ischemic injury, which is recognized in the gastrointestinal tract by the presence of denudation of superficial mucosal epithelium and loss of glands [15, 16]. In our case, the resection specimen showed all of the described features of TMA, with associated hemorrhage, ischemic injury, and ulceration. Subtle TMA features were present in the preceding colonic mucosal biopsies, but these were only identified on retrospective review.

There are a variety of reasons why pathology reports of mucosal biopsies may not include TMA in patients who are affected by the disorder. First, routine examination of GI mucosal biopsies typically includes evaluation of the epithelium and inflammatory cells of the lamina propria, with the intention of identifying neoplasms, inflammatory disease, or infectious processes. There is less focus on the microvasculature, since identification of disorders in this compartment is exceedingly rare. Thus, rare microthrombi may not be seen. In addition, seeing isolated thrombi in GI biopsies is not diagnostic of a systemic TMA by itself. If there is severe mucosal damage from any disease, few microvascular thrombi may develop in the immediate vicinity of the injury as a secondary process. The pathologist may interpret these few thrombi to be related to another disease in the biopsy and not include them in the report. Lastly, some cases of iTMA are best seen in the submucosa, where the most affected vessels exist, as in our case. Since GI biopsies only include the mucosa and possibly the very superficial portion of submucosa, the vessels with TMA may not be sampled. Thus, a GI biopsy report that is negative for TMA does not rule-out intestinal TMA. Given that TMA may be present at a higher rate in GI biopsies taken from patients with diarrhea following stem cell transplant, pathologists should carefully evaluate the microvasculature in this setting. In addition, it would be optimal for clinicians to notify pathologists of a possible TMA at the time of their examination and to encourage them to comment on the presence or absence of possible features of TMA in their report.

Our case adds to a body of evidence that suggests that criteria for TA-TMA might be improved by allowing for the possibility of an absence of schistocytes. While evidence for the use of eculizumab is limited in TA-TMA, this patient may have benefited from the medication had his condition been identified earlier [6, 21]. The role of TPE is less clear, as low ADAMTS13 does not play a role in the pathophysiology of TA-TMA while antibodies to complement factor H may be pathogenic in selected cases [22]. Most importantly, TA-TMA prevention via minimizing TA-TMA risk factors should be an integral part of stem cell transplant planning, as TA-TMA is frequently severe and lethal once occurs. At our institution, TA-TMA frequency decreased significantly with introducing post-transplant cyclophosphamide (PT-Cy) for GVHD prophylaxis (unpublished data). This could be explained by the fact that calcineurin inhibitors are introduced later at day + 5 in PT-Cy protocols versus at day − 1 or − 3 in standard GvHD prophylaxis protocols therefore avoiding overlapping endothelial damage from conditioning therapy with calcineurin inhibitor-induced endothelial damage.

## Conclusion

In summary, we describe a case of intestinal TA-TMA that occurred in the absence of peripheral blood schistocytes. Our experience underscores the importance of considering the diagnosis of intestinal TA-TMA in patients with refractory post-transplant diarrhea and GI bleeding, even if all classic features are not present. Future studies should aim to refine the diagnostic criteria for TA-TMA and to identify patients at highest risk for development of this condition.

## Abbreviations

TA-TMA: transplant-associated thrombotic microangiopathy
TMA: thrombotic microangiopathy
iTMA: intestinal thrombotic microangiopathy
aHUS: atypical hemolytic uremic syndrome
GVHD: graft versus host disease
GI GVHD: gastrointestinal graft versus host disease
HPF: high power field
SCT: stem cell transplant
CMV: cytomegalovirus
LDH: lactate dehydrogenase
INR: international normalized ratio
PTT: partial thromboplastin time
ICU: intensive care unit
ADAMTS13: a disintegrin and metalloproteinase with a thrombospondin type 1 motif, member 13
